# Sublingual allergen immunotherapy in immunosuppressed rheumatoid arthritis patients: Safety and clinical outcomes

**DOI:** 10.1016/j.jacig.2026.100728

**Published:** 2026-05-06

**Authors:** Yoshiaki Kobayashi, Daiki Nakagomi, Shunichiro Hanai, Nguyen Quoc Vuong Tran, Yuki Nakamura, Tomokazu Matsuoka, Daiju Sakurai, Atsuhito Nakao

**Affiliations:** a Department of Immunology, Faculty of Medicine, University of Yamanashi, Yamanashi, Japan; b Department of Rheumatology, Faculty of Medicine, University of Yamanashi, Yamanashi, Japan; c Department of Otorhinolaryngology, Head and Neck Surgery, Faculty of Medicine, University of Yamanashi, Yamanashi, Japan; d Atopy Research Center, Juntendo University School of Medicine, Tokyo, Japan

**Keywords:** Japanese cedar pollinosis, allergic rhinitis, sublingual immunotherapy, rheumatoid arthritis, IgE, IgG_4_, allergen immunotherapy

## Abstract

**Background:**

Allergen immunotherapy (AIT) is an effective disease-modifying treatment for allergic rhinitis. However, autoimmune diseases such as rheumatoid arthritis (RA) are traditionally considered relative contraindications, and evidence on the safety and efficacy of AIT in patients receiving immunosuppressive therapy remains limited.

**Objective:**

We assessed the clinical efficacy, safety, and immunologic effects of sublingual immunotherapy (SLIT) for Japanese cedar pollen (JCP)-induced allergic rhinitis in patients with RA well controlled by stable immunosuppressive treatment.

**Methods:**

Six patients with RA in remission who also had moderate-to-severe JCP-induced allergic rhinitis were enrolled. SLIT began in September 2024 and continued for 1 year. Clinical efficacy was evaluated by the Japanese Rhinoconjunctivitis Quality of Life Questionnaire and daily medication scores during the peak pollen seasons in March 2024 and March 2025. RA disease activity, adverse events, and JCP-specific IgE and IgG_4_ levels were measured every 3 months. Pollen counts were monitored using the Durham method at a regional environmental monitoring site.

**Results:**

Despite a higher pollen load in 2025 compared with 2024, symptom scores showed a tendency toward improvement, and quality-of-life outcomes suggested potential clinical benefit. No RA flares were observed, and immunosuppressive therapy was not modified. Immunologic analyses demonstrated changes in JCP-specific IgE and IgG_4_ consistent with SLIT exposure.

**Conclusion:**

In this small cohort, SLIT for JCP was well tolerated and associated with potential clinical and immunologic benefits in patients with stable RA receiving immunosuppressive therapy. These findings support the possibility that AIT may be feasible in selected patients with autoimmune diseases.

## Introduction

Allergen immunotherapy (AIT) is an effective disease-modifying treatment for allergic rhinitis (AR).^1^ However, autoimmune diseases, including rheumatoid arthritis (RA), have been regarded as relative contraindications for AIT.^2^ This caution arises from theoretical concerns that AIT-induced immune activation could exacerbate underlying autoimmune inflammation. In addition, in patients with autoimmune disorders, immune regulatory networks—such as regulatory T cell– and IL-10–mediated tolerance—are often dysregulated,^3^ making the immunologic response to AIT less predictable. Moreover, clinical evidence regarding the safety and efficacy of AIT in autoimmune populations remains extremely limited, as most clinical trials have excluded such patients. Therefore, current guidelines recommend that AIT be administered with caution or avoided in patients with autoimmune diseases.

Given the paucity of evidence on the efficacy of sublingual immunotherapy (SLIT) in patients with stable or well-controlled RA, this study evaluated the clinical efficacy, safety, and immunologic effects of SLIT for Japanese cedar pollen (JCP)-induced AR in patients whose RA remained stable with immunosuppressive therapy.

## Results and discussion

Six RA patients with moderate-to-severe JCP-induced AR were enrolled in January 2024. All participants demonstrated a serum JCP-specific IgE level of >0.35 UA/mL and moderate-to-severe nasal allergy symptoms, as defined by the Japanese guidelines for AR,^4^ for at least 1 week during each of the preceding two JCP dispersal seasons. RA was diagnosed according to the 2010 American College of Rheumatology/European League Against Rheumatism classification criteria. Patients with moderate or high RA activity or systemic corticosteroid receipt were excluded. At baseline, the disease of all patients was in remission according to 28-joint Disease Activity Score using erythrocyte sedimentation rate (DAS28-ESR) and Clinical Disease Activity Index (CDAI)^5^ and maintained stable immunosuppressive regimens (methotrexate, n = 4; salazosulfapyridine, n = 2; tocilizumab, n = 2) (Table I). Written informed consent was obtained, and the study was approved by the University of Yamanashi Ethics Committee (no. 2803).

**Table I:** Baseline characteristics of patients

Characteristic	Patient					
	A	B	C	D	E	F
Sex	Female	Male	Male	Male	Female	Female
Age (years)	63	48	68	56	56	39
Years with RA	5	18	4	9	11	7
Antibody of RA	RF, ACPA	—	ACPA	RF, ACPA	RF, ACPA	ACPA
Immunosuppressant for RA	TCZ	MTX	MTX	TCZ	MTX, SASP	MTX, SASP
Disease activity of RA	Remission	Remission	Remission	Remission	Remission	Remission
JCP-specific IgE (UA/mL)	46.6	3.27	2.78	9.25	9.61	5.05

*ACPA,* Anti-citrullinated peptide antibody; *MTX,* methotrexate; *RF,* rheumatoid factor; *SASP,* salazosulfapyridine; *TCZ,* tocilizumab.

SLIT was initiated in September 2024 and continued for 1 year. Adherence to SLIT was assessed by patient self-report and medication records, and all patients completed continuous treatment throughout the observation period. Patients received a fast-dissolving lyophilized JCP SLIT tablet (Cedarcure).^6^ Treatment followed the standard dosing regimen, consisting of an initial updosing phase of 2000 JAU administered over 1 week, followed by a maintenance dose of 5000 JAU once daily for the remainder of the study period. (JAU is a standardized allergen titer unit defined by the Japanese Society of Allergology.^7^)

RA activity was monitored with DAS28-ESR and CDAI, and SLIT-related adverse events were recorded every 3 months (September and December 2024; March, June, and September 2025). Serum JCP-specific IgE and IgG_4_ levels were measured on the same schedule using ImmunoCAP. Clinical efficacy was assessed in March 2024 and 2025, corresponding to the peak JCP season, using the Japanese Rhinoconjunctivitis Quality of Life Questionnaire (JRQLQ no. 1).^4^ Patients also recorded their daily receipt of oral antihistamines, nasal corticosteroids, and ocular antihistamines during March. Medication receipt was quantified as the rhinoconjunctivitis daily medication score, as previously described.^8^ For the medication score, one standard daily dose of an oral antihistamine (including fexofenadine hydrochloride, desloratadine, or bilastine) was assigned 6 points; one intranasal corticosteroid puff of mometasone furoate hydrate was assigned 1 point; and one ocular antihistamine drop of epinastine hydrochloride was assigned 1.5 points. Pollen counts were obtained by the Durham method from the Yamanashi Prefectural Institute for Health and Environmental Sciences. Exploratory paired comparisons between baseline and posttreatment season scores were performed by Wilcoxon signed-rank test. A 2-sided *P* value of <.05 was considered statistically significant.

**Fig 1:**
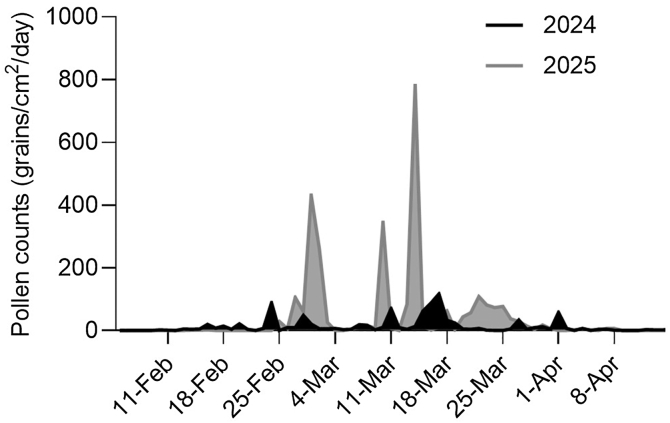
Comparison of daily JCP counts in 2024 *(black)* and 2025 *(gray).*

Despite nearly triple pollen exposure in 2025 compared with 2024 (2850.6 vs 960.3 grains/cm^2^) (Fig 1), nasal and ocular symptom scores in patients with RA showed a trend toward improvement compared with those in 2024, with reductions of approximately 30% to 60%. JRQLQ domains, including sleep and daily activity, also suggested consistent clinical improvement (Table II). The rhinoconjunctivitis daily medication score decreased during the 2025 season (Table II). Although most comparisons did not reach statistical significance owing to the small sample size, directional reductions in symptom and medication scores were observed.

**Table II:** Comparison of rhinoconjunctivitis symptoms, quality-of-life scores, and daily medication scores

Characteristic	Baseline (2024)	First season (2025)	Reduction∗	*P* value
Individual symptom score				
Runny nose	1.3 ± 0.5	0.7 ± 0.5	50%	.125
Sneezing	2.2 ± 1.0	0.8 ± 0.8	62%	.125
Nasal congestion	1.7 ± 1.2	1.0 ± 1.1	40%	.50
Itchy nose	1.7 ± 1.5	0.8 ± 0.8	50%	.25
Itchy eyes	2.0 ± 1.7	1.2 ± 1.0	42%	.25
Watery eyes	1.0 ± 1.3	0.7 ± 0.5	33%	.75
Individual component score				
Usual daily activities	0.9 ± 0.7	0.3 ± 0.6	68%	.03
Outdoor activities	1.2 ± 0.8	0.5 ± 1.0	57%	.25
Social functioning	0.8 ± 0.7	0.1 ± 0.1	94%	.12
Impairment sleeping	1.3 ± 0.8	0.3 ± 0.5	75%	.06
Physical problems	1.1 ± 1.2	0.5 ± 0.5	54%	.25
Emotional functions	0.9 ± 0.8	0.4 ± 0.3	63%	.12
General state	1.8 ± 1.0	1.5 ± 1.0	18%	.50
Daily medication score	7.0 ± 3.2	4.9 ± 3.2	31%	.125

Data are presented as means ± standard deviations. *P* values were calculated by Wilcoxon signed-rank test.

∗ Reduction was defined as difference between first season score relative to baseline score.

**Fig 2:**
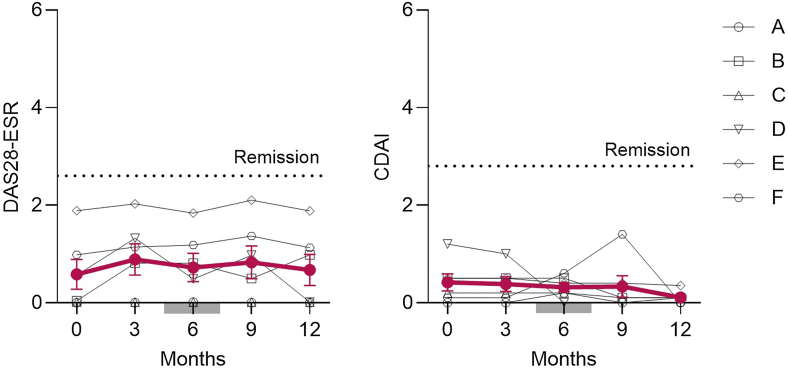
Changes in DAS28-ESR and CDAI scores every 3 months after SLIT. *Red bars* represent mean ± SEM values from 6 patients; *gray bar* indicates JCP dispersion period; and *dotted lines* represent remission cutoffs (DAS28-ESR 2.6; CDAI 2.8).

**Fig 3:**
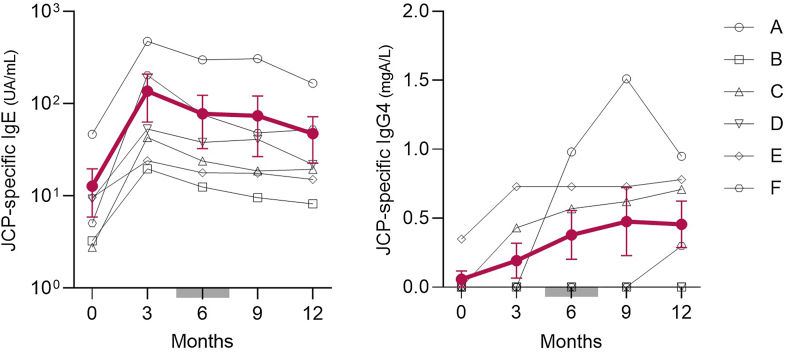
Changes in JCP-specific IgE and IgG_4_ levels every 3 months after SLIT. *Red bars* represent mean ± SEM values from 6 patients; *gray bar* indicates JCP dispersion period.

No patient experienced RA flare or required modification of immunosuppressive therapy. DAS28-ESR and CDAI remained stable throughout 1 year of SLIT (Fig 2). Adverse events were mild and local: 3 patients reported transient oral or throat discomfort, and one experienced brief laryngeal irritation, sneezing, and ocular itching; all resolved spontaneously without systemic treatment. No systemic allergic reactions or anaphylaxis occurred. Importantly, SLIT induced characteristic allergen-specific antibody changes (Fig 3). JCP-specific IgE increased transiently 3 months after the initiation of SLIT, followed by a blunted seasonal rise, while JCP-specific IgG_4_ progressively increased throughout the year.

In this small cohort, SLIT for JCP was well tolerated and associated with potential clinical and immunologic benefits in patients with RA in stable remission receiving immunosuppressive therapy. The kinetics of JCP-specific IgE and IgG_4_ were comparable to those observed in allergic individuals without RA.^6^

In this context, it is informative to consider how immunosuppressive therapies used in RA might influence the immunologic mechanisms underlying SLIT. Methotrexate broadly modulates immune responses across innate and adaptive immune compartments and reduces proinflammatory cytokine production such as IL-1, TNF-α, and IL-6, partly through adenosine-mediated mechanisms.^9^ Salazosulfapyridine also modulates inflammatory pathways,^10^ while tocilizumab suppresses IL-6–driven immune activation.^11^ Overall, these agents are generally considered to act on inflammatory pathways, whereas their direct effects on antigen-specific immune tolerance are less well defined. Our findings suggest that SLIT-induced immunologic modulation can be maintained despite concomitant immunosuppressive therapy, implying that inflammatory pathway suppression and antigen-specific tolerance induction may not be mutually exclusive processes. These observations further suggest that AIT may be applicable in patients with autoimmune diseases treated with similar immunomodulatory strategies and may help to identify immunosuppressive agents that are less likely to compromise the effectiveness of SLIT.

Our findings are consistent with a previous retrospective study demonstrating the safety and efficacy of AIT in 9 patients with various autoimmune diseases, including 5 patients with RA.^12^ We extend these observations by documenting allergen-specific antibody responses during ongoing immunosuppressive therapy. Moreover, by focusing exclusively on RA patients, with detailed medication tracking and quantitative assessment of pollen exposure, our study provides a more accurate evaluation of SLIT efficacy in this population.

In addition, a survey of members of the American Academy of Allergy, Asthma & Immunology reported a low frequency (2.8%) of serious adverse events during venom immunotherapy in patients with stable autoimmune disease, including RA.^13^ These observations are consistent with our findings and further support the feasibility of AIT in carefully selected patients. Accordingly, close collaboration between allergists and rheumatologists is important to ensure appropriate patient selection and coordinated management during SLIT.

Limitations include the small sample size, absence of a placebo control, and limited follow-up. Larger prospective studies with detailed immune profiling will help clarify how AIT interfaces with immune regulation in autoimmune disorders.

In summary, SLIT for JCP appeared to be well tolerated and associated with clinical improvement in patients with well-controlled RA receiving immunosuppressive therapy. These observations suggest that stable disease status may be an important consideration in patient selection. Although our findings are preliminary, they raise the possibility that the general relative contraindications for AIT in autoimmune disease may warrant reevaluation and could help inform future refinements of clinical guidelines.

**Key messages**

• SLIT for JCP appeared to be clinically beneficial and immunologically active in patients with stable RA receiving immunosuppressive therapy, without evidence of RA exacerbation.
• These results suggest that the traditional caution against AIT in autoimmune diseases may warrant reevaluation.

## Disclosure statement

Funded by a JSA WAO 2020 Memorial Research Grant Program to Y.K. and a Grant-in-Aid for Scientific Research to A.N. from the 10.13039/501100001700Ministry of Education, Culture, Sports, Science and Technology of Japan (grant 22K19427).

Disclosure of potential conflict of interest: The authors declare that they have no relevant conflicts of interest.
